# Acoustic Fractional Propagation in Terms of Porous Xerogel and Fractal Parameters

**DOI:** 10.3390/gels10010083

**Published:** 2024-01-22

**Authors:** Maria-Alexandra Paun, Vladimir-Alexandru Paun, Viorel-Puiu Paun

**Affiliations:** 1 Division Radio Monitoring and Equipment, Section Market Access and Conformity, Federal Office of Communications (OFCOM), 2501 Bienne, Switzerland; 2 Five Rescue Research Laboratory, 75004 Paris, France; vladimir.alexandru.paun@ieee.org; 3 Physics Department, Faculty of Applied Sciences, University Politehnica of Bucharest, 060042 Bucharest, Romania; viorel.paun@physics.pub.ro; 4 Academy of Romanian Scientists, 50085 Bucharest, Romania

**Keywords:** acoustic equation, fractional propagation, porous xerogel, SEM images, fractal parameters

## Abstract

This article portrays solid xerogel-type materials, based on chitosan, TEGylated phenothiazine, and TEG (tri-ethylene glycol), dotted with a large number of pores, that are effectively represented in their constitutive structure. They were assumed to be fractal geometrical entities and adjudged as such. The acoustic fractional propagation equation in a fractal porous media was successfully applied and solved with the help of Bessel functions. In addition, the fractal character was demonstrated by the produced fractal analysis, and it has been proven on the evaluated scanning electron microscopy (SEM) pictures of porous xerogel compounds. The fractal parameters (more precisely, the fractal dimension), the lacunarity, and the Hurst index were calculated with great accuracy.

## 1. Introduction

Porous substances comprise countless products made by human hands as well as genuine materials. In reality, all solid materials are porous to a certain degree, or on a certain measurement scale. However, the only famous genuine materials in preponderance that have a natural porosity high enough to have notable quantities of liquids or gases pass through them are soil/ground and rocks.

Porosity is defined as the ratio between the volume of voids amidst the solid particles of a material (solid substance) in its natural state and the total volume of the material. It is denoted by *p* and expressed as a decimal fraction or percentage. The current values of the porosity coefficient are generally distributed for some better-known materials such as the porosity of rocks in the natural layer. An example of this is between limestone or shale with a value of 0.10 or 0.01 (a minimum on the scale in terms of the nature of the layer) and clay with a value of 0.45–0.55 (the maximum), within the limits of the respective type of materials. The porosity of a material, being an intrinsic quantity, depends on the (effective) size of the granules, on the arrangement of the granules, on the non-uniformity coefficient, and on the shape of the granules [1,2,3].

Xerogel is a solid colloidal material obtained by drying a gel (e.g., gelatin or silica gel) at low temperatures (not higher than 100 °C), which is endowed with a consistent final structural shrinkage. Usually, a xerogel maintains a high porosity (which can reach 50%) and a huge specific surface area (150–900 m^2^/g), with very small pores (1–10 nm). On the other hand, when the liquid is removed under supercritical conditions, the structure does not tend to shrink and an aerogel is obtained, as a highly porous material (90%) with an extremely low density. We can also say that the sol–gel process is a method used to produce xerogels. By heating a xerogel at high temperatures, sintering of the porous gel occurs and ultimately becomes a dense glass.

The present paper provides information regarding how it has finally been possible to show that gas flow and liquid passage (and different other basic transport properties) also crossed other porous media such as those in the category of xerogels. The current presentation can optionally follow several theoretical paths. However, we will opt for only one of the ways, where the power–law distributions more correctly depicts the pore space and such distributions are congruent with the self-similarity connected with fractal or multi-fractal patterns [1,2,3]. The so-called fractal environment is an environment that benefits from the self-similarity quality regardless of the scale at which it is represented. However, numerous phenomena of real-world manifest restricted statistical or fractal properties [2]. But, it is not obligatory that these fractal objects have the same organization, no matter what the scale; they can be nearly self-similar, or they can be self-similar only for discrete values of the scale that appertain to a straight-line segment [*r_min_, r_max_*], where *r_min_* (*r_max_*) is the smallest (the largest) scale value present in the considered geometrical object [3].

In this article, the advanced continuum-type mechanics of porous media with a generally anisotropic, mathematical-product-like fractal geometry are considered. Relying on a fractal derivative, the approach leads to global balance laws in terms of fractal functions and their derivatives. This is based on product measures and then converting them to integer-order differential equations in conventional (Euclidean) space. The theoretical strategy, in the last instance, follows the approach to continuum-type mechanics of heterogeneous porous media of the fractal type. The final objective is to develop a continuum physics-type framework for the diffusion of liquids and gases in elastic–inelastic deformable solid porous materials. More precisely, the reasonable association of this type of behavior with an acoustic fractional propagation equation was successfully achieved.

Another technique for solving fractional differential equations is that based on the introduction and implementation of an optimal auxiliary function method to solve a system of fractional-order Whitham–Broer–Kaup equations; these are a class of nonlinear partial differential equations with broad applications in mathematical physics. This method provides a systematic and efficient approach to finding accurate solutions for complex systems of fractional-order equations [4,5,6].

The fractal environment can be fashioned as mensurable metric assortments, each having non-integer dimensions [7,8,9,10,11]. The non-integer Hausdorff dimension [7] is commonplace in fractal theory and also a major property. The isotropic fractal material is distinguished by the mass, *M*, relation of a ball region, *V*, of fractal material, *M* = *M*(*V*), which is function of $M_{0}$, *R*, and $R_{0}$. This is basically the most general connection between them. More precisely:

(1) $$M(V)=M_{0}{\left(\frac{R}{R_{0}}\right)}^{D}$$

where $R_{0}$ characterizes the associated size of the fractal environments [7,8] and *D* is the mass measure of the fractal environment.

The anisotropy property of fractal materials can be taken into consideration utilizing a power–law formula, for the mass of a parallelepiped section *Vp* of the studied object, in the present mathematical form:

(2) $$M(V_{p})=M_{0}{\left(\frac{L_{x}}{R_{0}}\right)}^{\alpha_{1}}{\left(\frac{L_{y}}{R_{0}}\right)}^{\alpha_{2}}{\left(\frac{L_{z}}{R_{0}}\right)}^{\alpha_{3}},\;\mathrm{with}\;min\left\{L_{x},\;L_{y},L_{z}\right\}\gg R_{0}$$

where $\alpha_{i}$ are non-integer values for the mathematical dimension on the coordinate axes $X_{i}$ and i=1,2,3 are counting pointers. In this context $L_{x},L_{y}$, and $L_{z}$ are the three edges meeting at the same vortex. The parameter $\alpha_{i}$ delineates/relates to the expansion in the mass of this considered environment when one of parallelepiped dimensions is enlarged along one axis, and the other dimensions of the parallelepiped along the other axes are not modified. Then, the expression *D =* $\alpha_{1}$ *+* $\alpha_{2}$ *+* $\alpha_{3}$ represents the fractal mass size of the anisotropic fractal environment. Thus, we can now define fractal material as an environment that has a non-integer mass dimension, according to Equations (1) and (2). Though the non-integer size does not completely depict all of the physics aspects of the fractal environment, it is considered a relevant property of fractal material [8].

This paper includes a consistent introduction in Section 1. This introduction presents xerogels and their particular porosity, together with their fractal behavior. Section 2 is titled 2. Results and Discussion. In Section 2.1, fractal parameters including the fractal dimension, the lacunarity concept, and the Hurst exponent are introduced, in order to be able to understand the notions of the particular morphology of the porous xerogels considered in continuation. In Section 2.2, the fractional differential equation of acoustic wave propagation in a fractal porous media is established and solved in relation to the dynamics of gas movement through xerogel porous media. Circulation is primarily due to the presence of pores in the material and its fractal nature. Section 2.3 includes the results and discussion, referring to the results obtained by studying the structure of the SEM-investigated samples. In addition, it presents the calculated values of the associated fractal parameters. Section 3 is dedicated to presenting the conclusions of this article. Finally, Section 4 details the materials and methods, with the xerogel materials and synthesis in Section 4.1, the xerogel characterization in Section 4.2, and the morphology in Section 4.3.

## 2. Results and Discussion

### 2.1. Fractal Parameters

In terms of fractal assumption of natural reality, the fractal parameters are the most important theoretical components. Of these, the most widely used are the fractal dimension, the lacunarity, the succolarity concept, and the Hurst exponent, being fluently utilized to permit access to the structural circumstances of porous-material intricate assessment in reciprocation [7,8,9,10,11]. However, we now specify that succolarity is less necessary for evaluating the fractal aspect of the behavior of porous materials. This was the reason for not using it, as it was practically useless on this occasion. The realistic application and correct valuation of the three aforementioned necessary parameters are arduous, due to the complicated definitions and laborious procedures of calculus [9]. In the following section, we introduce the accredited definitions, the substantial significance of the fractal characteristics, and the evaluation procedure based on the box-counting method from images in principle, but on alternative practices too.

#### 2.1.1. Fractal Dimension

A fractal object can be defined taking into account the fact that its fragments have an identical/self-same look with the entirety, that is, they are geometrically similar even if subsequent simple modifications are applied. In addition, if the fractal dimension is made up of a certain number of duplicates, each transformed to a determined scale and eventually slightly remodeled, this will be named a self-similar object. The self-similarity quality is really important and nearly all natural objects are capable of being self-similar. Projective transformations, which are defined as combinations of translating, and rotations of space, together with scaling operations, can be used to express the changes that the entire object passes through to get into the new shape. Hereafter, we will refer to the most familiar method of calculating the fractal dimension as the box-counting method.

To calculate the fractal dimension by the box-counting dimension, the considered figures must be segregated into quadrats (2D) or cubes (3D), with a side/edge length of *ε* (1D). To completely comprise the fractal geometrical object, only the same type of cassettes are utilized. These boxes, which overlap the measured initial geometrical object, will have a supposed *N* complete number.

Note: The total length value of the measured curve undergoes an essential conversion according to each ruler of scale used, and the maximum number of boxes differs consistently, as the size of each box undergoes modifications.

The meaning of the box-counting dimension, Db, is the ratio limit of logN(ε) and log(1/ε), in the situation in which ε (the length) drives/tends to zero, such as in the expression:

(3) $$Db=\underset{\varepsilon \to 0}{\lim}\frac{logN(\varepsilon )}{\;log(1/\varepsilon )}=-\underset{\varepsilon \to 0}{\lim}\frac{logN(\varepsilon )}{log\varepsilon }$$

Conformable to the box (cassettes) numeration technique and viewed as an easily accessible global measurement, the box-counting additive routine [6,9] can be assumed as being precious both for simulated fractals as well as genuine fractals, indifferent of their degree of complexity.

#### 2.1.2. Lacunarity

A good lacunarity definition is the one that determines with certainty, first of all, the gap size, or the pore distribution in the considered corpus. Furthermore, it takes into consideration the volume of real holes and apportions them without physical content matter, in comparison to the total accessible volume [8]. If the geometrical body also has major holes or defects, (in the sense of being lacunar), the more likely its voids are to be larger, due to the included special open formal figures, such as disks (2D) or spheres (3D). Thus, for a fractal object, the more numerous the defects or holes (i.e., presenting more lacuna), the bigger the lacunarity is considered to be. On the other hand, lacunarity evaluated as small demonstrates a superior homogeneity value in terms of the assessed images [7,8,9].

Equation (4) Λ(*ε*) is the lacunarity value and *ε* is the box dimension dataset [7,9].

(4) $$\Lambda \left(\varepsilon \right)=\frac{Z^{\left(2\right)}}{{\left(Z^{\left(1\right)}\right)}^{2}}$$

$Z^{\left(1\right)}\;and\;Z^{\left(2\right)}$ represent the first and second moments, respectively:

(5) $$Z^{\left(1\right)}=\sum_{\varepsilon }P•Q\left(P,\varepsilon \right)=\sum_{\varepsilon }P•\frac{n(P,\varepsilon )}{{\left(M-\varepsilon +1\right)}^{2}}$$

and

(6) $$Z^{\left(2\right)}=\sum_{\varepsilon }P^{2}•Q\left(P,\varepsilon \right)=\sum_{\varepsilon }P^{2}•\frac{n(P,\varepsilon )}{{\left(M-\varepsilon +1\right)}^{2}}$$

In Equations (5) and (6), *M* is the map dimension and *P* is equal to the box mass. The *n*(*P*,*ε*) is the box number that contains *P* pixels, and *Q*(*P*,*ε*) is the statistical probability.

#### 2.1.3. Hurst Exponent Evaluation

The Hurst index (exponent), *H*, depicts the motion roughness. The smaller the value, the greater the roughness and vice versa. When the *H* index is equal to 0.5, the movement is declared to be a Brownian process, and when it is much smaller than 0.5, there is a growing incremental negational correlation (very craggy movement). When it is greater than 0.5, a positive correlation of the incrementals occurs (flattened movement). The fractal dimension (*D*) and the Hurst exponent (*H*) of the fractal identical object are linearly related by the formula *D* = 2 − *H*, where 0 ≤ *H* ≤ 1 and 1 ≤ *D* ≤ 2. The Hurst exponent can be immediately calculated with the expression *H* = 2 − *D*, using the fractal dimension computed with the box-counting method [10,11].

#### 2.1.4. Implications of the Calculated Fractal Parameters on the Properties or Behaviors of the Xerogel-Type Materials

The fractal dimension quantifies the change in the mass of solid as a function of the observation scale. In addition, the fractal dimensions exhibit the evaluated surface roughness degree. It is known that the strength of xerogels is increased by adding additives, and the shrinkage is also restrained in the course of drying. The calculated fractal dimensions and the thermodynamic correlations have almost the same tendencies. Consequently, the increase in strength with the application of additives and the restriction of shrinkage with the drying process are precisely reflected in the fractal dimension values. Moreover, the higher fractal dimension could also be due to a change in the microstructure happening during the aging or drying process, which could increase the connectivity. 

A lacunarity intuitional definition refers to the fact that it is a good measure of the distribution of crevasses (gaps). More precisely, it is a reflection of the volume of gaps/holes (the portions without physical material present) when compared to the entire available volume. In the fractality assumption, the lacunarity concept relates to and defines/explains (mathematically) the presence of current holes (that is why it is named after a porous texture). On top of that, we find it to be synonymous with the quantitative radiography of the “correct or real texture” [7,8].

Note: The fractal dimension is a little smaller, but the lacunarity is greater due to the fact that the xerogels used in the current study have more voids/interstices, such that they are able to diffuse the transmitted gases or liquids in larger quantities and faster.

### 2.2. Fractional Propagation Equation in Fractal Porous Media

The acoustic fractional propagation equation [12,13,14,15,16] in a fractal porous media is given by:

(7) $$\frac{\partial^{2}p}{\partial x^{2}}\left(x,t\right)+\left(\frac{1-\alpha }{x}\right)\frac{\partial p}{\partial x}\left(x,t\right)-\left(\frac{\Theta^{2}\left(\alpha \right){\left|x\right|}^{2\alpha -2}}{c^{2}}\right)\frac{\partial^{2}p}{\partial t^{2}}\left(x,t\right)-B\Theta^{2}\left(\alpha \right){\left|x\right|}^{2\alpha -2}\frac{\partial^{\frac{3}{2}}p\left(x,t\right)}{{\partial t}^{\frac{3}{2}}}=0$$

where *p* = *p*(*x*,*t*) is the acoustic field pressure, and:

(8) $$\Theta \left(\alpha \right)=\frac{\pi^{\frac{\alpha }{2}}}{\Gamma \left(\frac{\alpha }{2}\right)}$$

In Equation (8), $\left(\alpha \right)=(\alpha_{1,}\alpha_{2,}\alpha_{3,})$ is the multi-index and $\Gamma \left(x\right)$ is the gamma function [17]. In the fractal porous environment, the wave-front velocity is:

(9) $$c^{\prime }=\frac{c}{\Theta \left(\alpha \right)}{\left|x\right|}^{1-\alpha }=(\frac{c}{\pi^{\frac{\alpha }{2}}}){\Gamma \left(\frac{\alpha }{2}\right)\left|L\right|}^{1-\alpha }$$

which highlights the fact that the wave-front speed in the fractal media is of a self-similar type. In Equation (9), *L* is correlated to the thickness value of material and *c* is the wave-front velocity in a non-fractal (without the self-similarity property) porous media [12,13].

**Figure 1:**
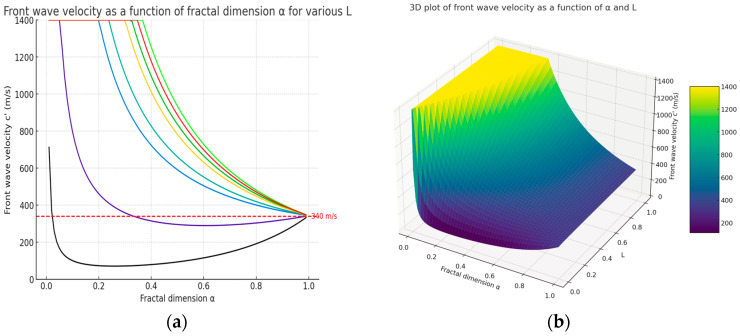
Wave-front velocity in the fractal media as: (**a**) a fractal dimension function for various *L* (2D); (**b**) a function of fractal dimension and *L* (3D).

In Figure 1a, we present the computed wave-front velocity (*c′*) as a function of the fractal dimension α for various levels of the parameter *L*, ranging from 0.01 to 1.0. In Figure 1b, the 3D surface plot illustrates the variation in the wave-front velocity (*c′*) as a function of the fractal dimension α and the parameter *L*. The fractal dimension α is represented along the x-axis, and *L* is depicted on the y-axis, with both variables ranging from 0.01 to 0.99 to avoid singularities at the extremes.

Each curve is drawn with a color; the first black curve (from bottom to top) is for the *L* = 0.01 value and the last light green curve is for the *L* = 1 thickness value. A horizontal reference line is drawn at 340 m/s, representing the sound speed in air at room temperature, to constitute a threshold of interest. This is possibly related to a physical phenomenon under investigation. This visualization supports our analysis of fractal material behavior, particularly in terms of how the fractal dimension influences the wave propagation speed in such media.

Note: For very low values of fractal dimension, the wave-front velocity tends to high values, which is due to the mathematical properties of the function Γ(*x*), when *x* tends to the zero limit. For *L* values greater than *L* = 0.3 m, we observe that the wavefront velocity increases rapidly with the fractal dimension, touching supersonic values, and all being located above the dotted horizontal reference line. Considering that, in traditional non-fractal porous materials, the wavefront speed is always lower than the speed of sound in air, we still see here that the wavefront speed can be higher than the speed of sound in air (supersonic speed) for porous fractal materials. This is an interesting novelty for the porous xerogels comportment to take into account.

#### Solution of Fractional Propagation Equation in Fractal Porous Media

In the current section, we will attempt to resolve propagation Equation (7) in the Laplace domain, utilizing the following initial conditions:

(10) $${\left.p\left(x,t\right)\right|}_{t=0}={\left.\frac{\partial p}{\partial t}\left(x,t\right)\right|}_{t=0}=0,$$

We note $P\left(x,z\right)$ and the Laplace transform of $p\left(x,t\right)$ is defined by:

(11) $$P\left(x,z\right)=L\left[p(x,t)\right]=\int_{0}^{\infty }\exp\left(-zt\right)p\left(x,t\right)dt.$$

The Laplace transform L of $p\left(x,t\right)$, Equation (7) becomes:

(12) $$\frac{1}{W(\alpha ,x)}\frac{\partial }{\partial x}\left(\frac{1}{W\left(\alpha ,x\right)}\frac{\partial P}{\partial x}(x,z)\right)-k^{2}\left(z\right)P\left(x,z\right)=0.$$

where:

(13) $$k^{2}\left(z\right)=\frac{z}{c^{2}}\left(z+b^{\prime }\sqrt{z}\right),\;b^{\prime }=Bc^{2}\sqrt{\pi }.$$

In Equation (12), W(α,x) is the fractional density of states defined by the measure for integration in non-integer dimensional space, or a fractal space. It is defined by the following expression:

(14) $$W\left(\alpha_{k},x_{k}\right)=\frac{\pi^{\alpha_{k}/2}}{\Gamma \left(\alpha_{k}/2\right)}{\left|x_{k}\right|}^{\alpha_{k}-1}$$

By substituting the expression of W(α,x) in Equation (12) through Equation (14), we acquire:

(15) $$\frac{\partial^{2}P\left(x,z\right)}{\partial x^{2}}+\left(\frac{1-\alpha }{x}\right)\frac{\partial P}{\partial x}\left(x,z\right)-k^{2}\left(z\right)\Theta^{2}\left(\alpha \right)x^{2\alpha -2}P(x,z)=0$$

To determine the solution to Equation (15), we proceed to the function change: $P(x,z)=x^{\nu }y$, where ν is a parameter to be determined [14,15]. Equation (12) becomes:

(16) $$x^{2}y^{\prime \prime }+(2\nu +1-\alpha )xy^{\prime }+\left[\nu \left(\nu -1\right)+\left(1-\alpha \right)\nu -k^{2}\Theta^{2}x^{2\alpha }\right]y=0,$$

By taking ν=α/2, Equation (16) is reduced to:

(17) $$x^{2}y^{\prime \prime }+(2\nu +1-\alpha )xy^{\prime }+\left[\nu \left(\nu -1\right)+\left(1-\alpha \right)\nu -k^{2}\Theta^{2}x^{2\alpha }\right]y=0,$$

which is transmuted by the variable replacement of $u=k\left(z\right)\Theta \left(\alpha \right)x^{2\nu }/2\nu$ into:

(18) $$u^{2}+uy^{\prime }-\left(u^{2}+\frac{1}{4}\right)y=0,$$

which is the Bessel differential equation. The general solution is:

(19) $$y\left(u\right)=a\left(z\right)I_{\frac{1}{2}}\left(u\right)+b\left(z\right)K_{\frac{1}{2}}(u),$$

with $I_{\frac{1}{2}}$ and $K_{\frac{1}{2}}$ being the modified Bessel functions of index ½. 

The solutions of Equation (15) can now be written as:

(20) $$P\left(x,z\right)=\sqrt{\frac{2}{\pi }}a\left(z\right)\exp\left\{\frac{k\left(z\right)\Theta \left(2\nu \right)x^{2\nu }}{2\nu }\right\}+\sqrt{\frac{\pi }{2}}a\left(z\right)exp\left\{\frac{k\left(z\right)\Theta \left(2\nu \right)x^{2\nu }}{2\nu }\right\}$$

where $a\left(z\right)$ is a new constant [16].

From Equation (17), where the condition $k^{2}\left(z\right)\Theta^{2}\left(\alpha \right)$ = 1 is fulfilled and the parameter to be determined is equal to ν=1/2, we now obtain:

(21) $$x^{2}y^{\prime \prime }+xy^{\prime }-(x^{2}+\frac{1}{4})y=0.$$

Equation (21) is a special case of the zero-order Bessel’s equation. For positive values of the argument *x*, that is, for *x* > 0, the general solution y of the Bessel equation of zero order is a linear combination of zero-order Bessel functions, *J*_0_(*x*) and *Y*_0_(*x*):

(22) $$y={c_{1}J}_{0}\left(x\right)+{c_{2}Y}_{0}\left(x\right)$$

**Figure 2:**
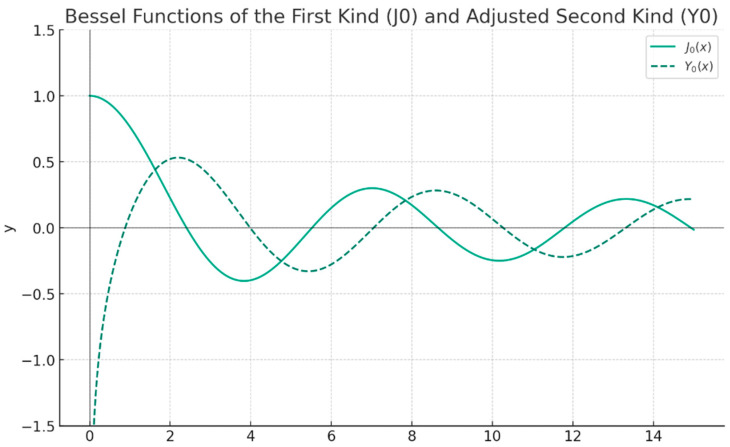
The Bessel functions of zero order.

The graphic representation of the Bessel functions of zero order, *J*_0_(*x*) and *Y*_0_(*x*), is shown in Figure 2.

Note: It is easily observed that $\underset{x\to 0}{\lim}J_{0}\left(x\right)=1$ and that $Y_{0}\left(x\right)$ has in *x* = 0 a singularity of a logarithmic type. Thereby, if we are concerned with Bessel’s equation solutions of zero order that in origin have finite values, which is frequently the mathematic cover, it is necessary to disclaim *Y*_0_. The graphical represents of the Bessel functions *J*_0_ and *Y*_0_ are introduced in Figure 2. It is extremely interesting to observe from the cited figure that for *x* high, the functions *J*_0_(*x*) and *Y*_0_(*x*) have an oscillatory behavior. Still, such a comportment might be anticipated, even from the original equation [16]. 

In the case where the condition k^2^ (z) Θ^2^ (α) = 1 is fulfilled and the parameter is equal to ν = 1/2, the Equation (21) becomes:

(23) $$x^{2}y^{\prime \prime }+xy^{\prime }-\left(x^{2}+1/4\right)y=0.$$

By dividing both members by *x*^2^, Equation (23) is obtained:

(24) $$y^{\prime \prime }+(\frac{1}{x})y^{\prime }-\left(1+\frac{1}{4x^{2}}\right)y=0.$$

When *x* is very large, it is more than reasonable to consider that the terms (1/x)$y^{\prime }$ and ($\frac{1}{4x^{2}}$) y are small (practically both tend to zero) and can be neglected. It is therefore logical to admit that Equation (23) can now be approximated with another:

(25) $$y^{\prime \prime }-y=0.$$

This valuable fact is correct to the extent that the Bessel functions are of the oscillatory type; but, this is only true to a certain extent.

For large a *x* value, the functions $J_{0}$ and $Y_{0}$ also decay as *x* increases. Thus, the equation *y″* + *y* = 0 does not provide an adequate approximation to the Bessel equation for a large *x*, and a more delicate analysis is required. For a considerably high *x*, the functions $J_{0}$ and $Y_{0}$ also decline as *x* augments. It is simply shown that the solutions of this differential equation are sin *x* and cos *x*. More precisely, the solutions of Bessel’s equation for several values of *x* are identical to the linear combinations of the sin *x* and cos *x* functions. Without discussion, it can be shown simply that:

(26) $$J_{0}\left(x\right)≅{\left(\frac{2}{\pi x}\right)}^{\frac{1}{2}}\cos\left(x-\frac{\pi }{4}\right)\;as\;x\to \infty$$

together with:

(27) $$Y_{0}\left(x\right)≅{\left(\frac{2}{\pi x}\right)}^{\frac{1}{2}}\sin\left(x-\frac{\pi }{4}\right)\;as\;x\to \infty$$

Note: Let $y_{1}\left(x\right)$ be the solution of differential Equation (21), with:

(28) $$y_{1}\left(x\right)=a_{0}\left[1+\sum_{m=1}^{\infty }\frac{{\left(-1\right)}^{m}x^{2m}}{2^{2m}({m!)}^{2}}\right],\;x>0.$$

**Figure 3:**
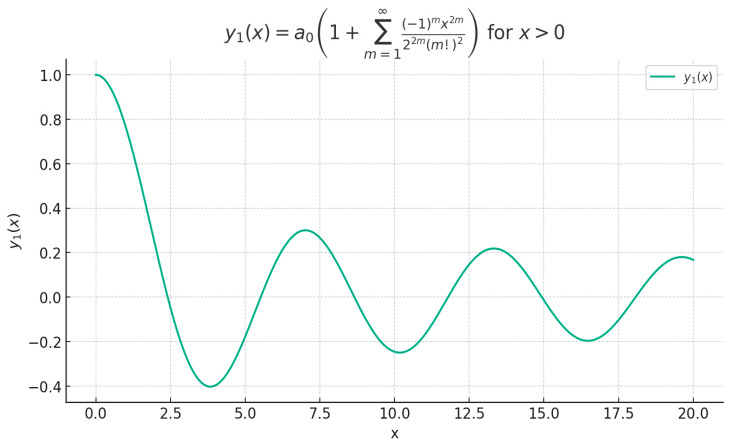
The polynomial solution $y_{1}\left(x\right)$ of differential Equation (28).

Figure 3 represents a polynomial approximation to the *J*_0_(*x*) function by means of the function *y*_1_(*x*), which is the solution of differential Equation (28). 

**Figure 4:**
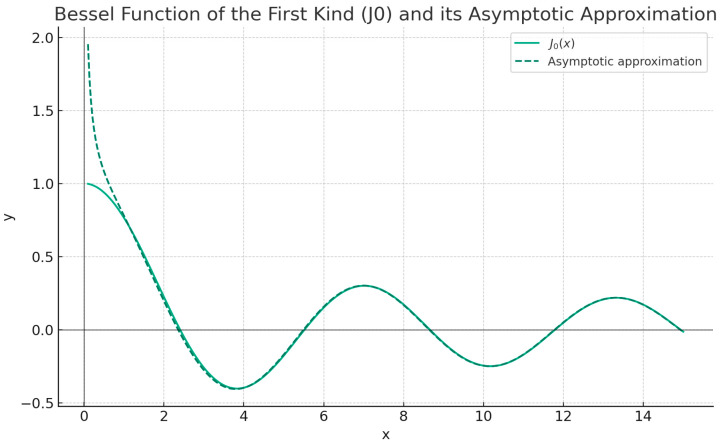
Graphic representation of $J_{0}\left(x\right)$. Asymptotic approximation.

It is easy to show that Equation (26) is convergent no matter what the argument *x* is and that $J_{0}$ is analytic in *x* = 0. In Figure 4, one can see the graphs of the function ${y=J}_{0}\left(x\right)$ along with some of the partial sums of the series under discussion.

Figure 4 represents the solution of the Bessel differential Equation (25).

It can be said that these asymptotic approximations, such as *x* → ∞ are, in truth, very good. For instance, in Figure 4, it looks as though the asymptotic approximation (26) for $J_{0}\left(x\right)$ is naturally precise for all *x* ≥ 1. However, to approximate $J_{0}\left(x\right)$ over the entire positive domain, in the range from zero to infinity, we can utilize two or three terms of the series (26) for *x* ≤ 1 and the asymptotic approximation (28) for *x* ≥ 1.

### 2.3. Fractal Analysis

In order to carry out fair comparisons and exemplary discussions in which usual xerogels are involved, some chitosan xerogel explanation samples were processed in the analogous conditions as the hydrogels mentioned in Section 4; all were denoted with the letter F and a certain number of indices [18,19,20]. In other words, we have obtained the SEM experimentally realized images of several different xerogels, each having diverse degrees of porosity [21]. In the following section, however, we will present only the picture that proved to be the most representative [22,23,24].

#### Fractal Analysis of SEM Picture

**Figure 5:**
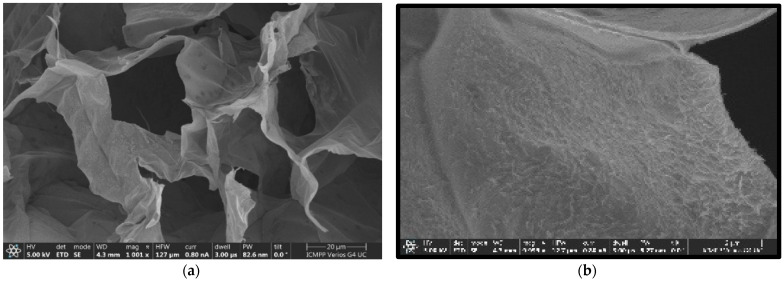
(**a**) Original SEM image with the material pores evidenced; (**b**) Indoor shot enlarged 5 times compared to the original.

The SEM picture scale bar/line, on the lower right side of Figure 5a, has a length of 100 microns and 1001× magnification. The value of the high-voltage power supply (HV) is 5 kV [25,26]. The second image, Figure 5b, benefits from a magnification that is five times higher than the first image. We can practically say that we captured a portion of a pore together with its wall. 

**Figure 6:**
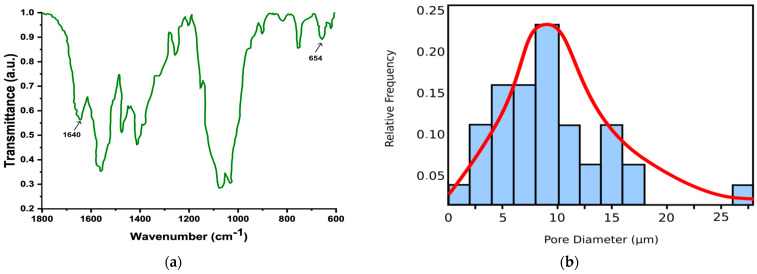
(**a**) Simple xerogel FTIR spectra; (**b**) Statistical histogram to pore size distribution of L SEM picture.

In Figure 6a the FTIR (Fourier transform infrared spectroscopy) spectra of simple xerogel, coded F, is shown, and in Figure 6b, the statistical pore histogram of the L SEM picture is represented. 

Fourier transform infrared spectroscopy (FTIR) is a technique used to obtain an infrared spectrum of the absorption or emission of a solid, liquid, or gas. FTIR spectra were produced using a Bruker device, vector 22 (Ettlingen, Germany) at room temperature with a wavenumber resolution of 1 cm^−1^ using KBr pellets in the frequency range of 4000–400 cm^−1^. In addition, to obtain a good signal-to-noise ratio, 32 scans were run and averaged. Figure 6a shows the obtained FTIR spectra.

The energy-dispersive X-ray analyzer (EDX) has been utilized for the identification of the individual chemical elements in the examined composition for the identification of the investigated xerogel, such as that in Figure 6a. More precisely, in this figure, an electromagnetic emission spectrum is presented. On the Ox-axis (abscissa), the emission energy, is found, representing the energy at which the atoms emit X-rays whenever they enter into an interaction with an electron flow. The respective energy is well established, being the basic characteristic of every atom, and the recorded spectrum marks the presence of these specific values in the evaluated material sample. The counts are compiled on the Oy-axis (ordinate). In principle, EDX analysis is an elemental surface investigation, its purpose being to establish with certainty the categories of atoms that exist in a tested specimen. The histograms of Figure 6b present the pore dimension distribution data obtained from the F-index SEM image. Concerning the xerogel histogram from the L SEM image, we can say that its upper limit is a normal (Gaussian) distribution, that is, the continuous red line in the graph.

**Figure 7:**
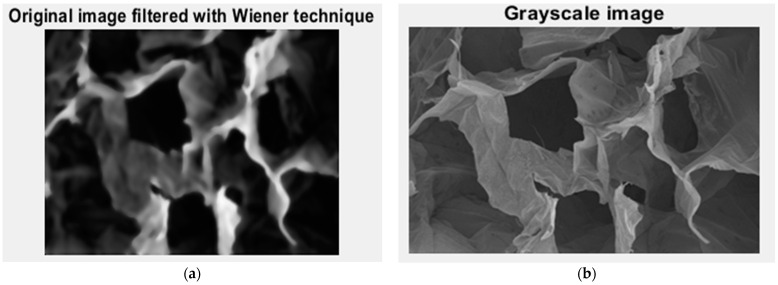
(**a**) The filtered version of the SEM image; (**b**) The processed grayscale image.

In Figure 7a, the initial image filtered with the Wiener technique is found, and in Figure 7b, the image in grayscale version is represented.

**Figure 8:**
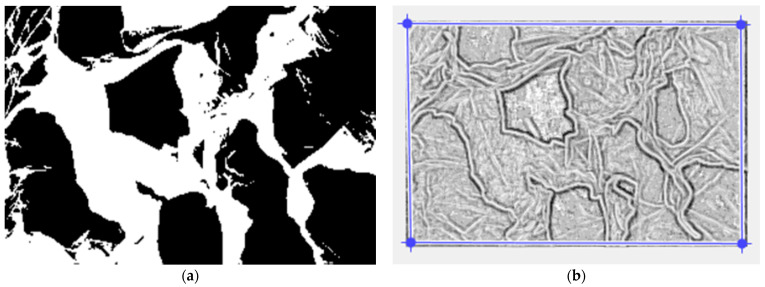
(**a**) The binary version of the picture; (**b**) The mask made for the lacunarity calculation.

Figure 8a,b present the pictures prepared in the binary version and the fixing of the applied mask, respectively, utilized to compute the lacunarity.

**Figure 9:**
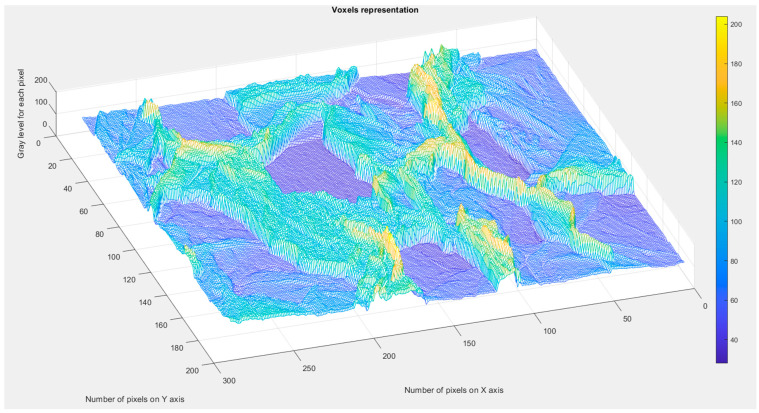
Voxels representation within the evaluated image.

Figure 9 presents the assessed voxels of the L picture, which is a 3D eloquent voxels portrait of the gray-level amount on the Oz-axis. This conforms with the position and number of pixels, labeled on the last two plane axes: the Ox-axis and Oy-axis, respectively.

**Figure 10:**
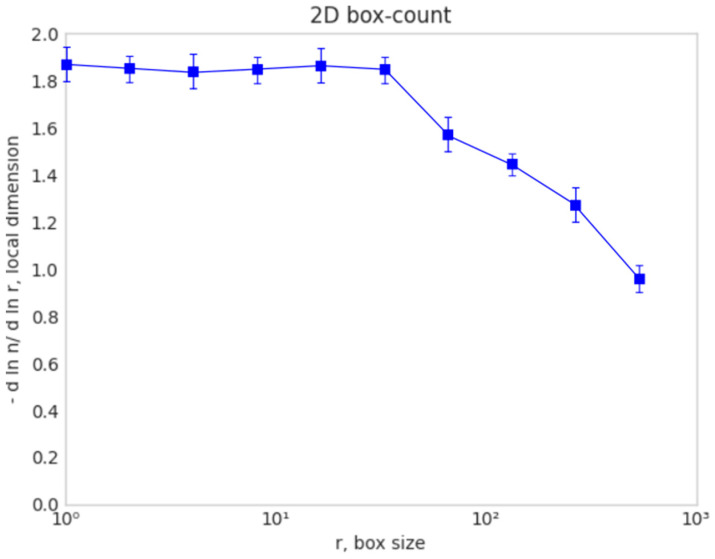
Local fractal dimension evaluated by the box-counting method.

Figure 10 shows an examination of the elected image area; more precisely, this figure shows the fractal dimension calculation as the box size *r* function with dedicated software for the box-counting procedure. The blue vertical bars attached to each point (intersecting the horizontal line) represent the standard deviation (square root of the variance).

Basically, we have the local fractal dimension computed with the 2D box-computing method.

**Figure 11:**
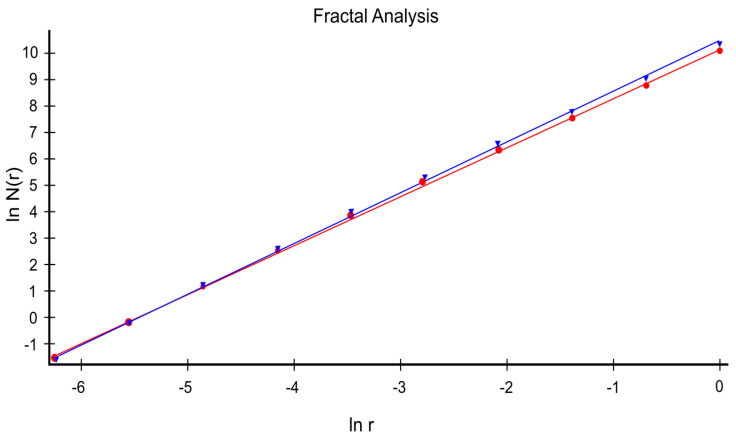
Verification of the results using the HarFA program.

Figure 11 presents the straight lines achieved by implementing multiple linear regression to certain accurate data collections, considered as checking the function ln(*N*(*n*(*r*)) depending on the argument ln(*r*).

Statistics

Blue regression line: y = 1.9063x + 10.4307, R = 9.99951

Red regression line: y = 1.8503x + 10.1113, R = 0.99939

In Figure 11, the slopes of the straight blue and red lines achieved through multiple linear regression are equal to the suitable fractal dimension of the considered SEM picture [18]. Thus, the fractal dimension values of *d*_1_ = 1.850 for the red regression line (square mask) and *d*_2_ = 1.906 for the blue regression line (rectangular mask) were obtained. The Hurst index (exponent) *H* = 2 − *D* calculated with a square mask is *H*_1_ = 0.150 and calculated for the type of rectangular mask is *H*_2_ = 0.094.

For the same xerogel SEM image, the fractal parameter values are found in Table 1. 

Table 1 is founded on the fractal parameter values achieved by the complex processing of the original SEM image. In this table, *FD*_1_ is the fractal dimension calculated with a square mask, *FD*_2_ is the fractal dimension calculated with a rectangular mask, *SD*_1_ is the standard deviation calculated with a square mask, while *SD*_2_ is the standard deviation of the fractal dimension calculated for the type of rectangular mask. 

The specific fractal parameters calculated in this study are the fractal dimension, the Hurst index, and lacunarity [27,28]. The high accuracy with which these fractal indicators were calculated is owing to the methods and the advanced computational software used. Thus, the box-counting method was used to calculate the fractal dimension (see Figure 10), with different algorithms for a square mask and rectangular mask. As a measure of the particular accuracy obtained, they stand as testimony for the values of standard deviation *SD*_1_ calculated with a square mask [29] and the standard deviation of the fractal dimension calculated for the type of rectangular mask *SD*_2_ [30]. 

To calculate the lacunarity, the modified SEM figures were used, with the binary version of the picture and the mask with well-defined pore contours (see Figure 8a,b). Finally, Figure 9 presents the assessed voxels of the SEM picture with a 3D eloquent voxels portrait of the gray-level amount on the Oz-axis, conforming with the position and number of pixels, labeled on the last two plane axes: the Ox axis and Oy axis, respectively. In fact, it shows the 3D vision of the lacunarity due to the clear distinction of the pores in a processed SEM image.

The numerical assessment upshot of the elected F SEM image, effectuated by the fractal characterization software cultivated by the authors, is the fractal dimensional worth *FD*_1_ = 1.668 and *FD*_2_ = 1.615, standard deviation worth *SD*_1_ = ±√(σ^2) = ±0.3127 and *SD*_2_ = ±√(σ^2) = ±0.1445, and lacunarity worth *Λ* = 0.0526. These are presented in Table 1. The Hurst index (exponent) *H* = 2 − *D* calculated with a square mask is *H*_1_ = 0.332 and calculated for the type of rectangular mask is *H*_2_ = 0.242, [10,11].

Finally, we will carry out a comparison of the obtained results in this paper with those published in the only two papers we know of, belonging to us, where a complete analysis of the morphology details of xerogels using multifractal analysis and scanning electron microscopy images was carried out [31] (see the bibliography). In the first paper, the fractal parameters of the SEM images of 5-fluorouracil released from a chitosan-based matrix were evaluated. The average values were for a fractal dimension of *D* = 1.8621 ± 0.0733 and a lacunarity value of *Λ*_1_ = 0.0385 [31]. In the second article, the average value for the fractal dimension of a rectangular mask is *D*_1_ = 1.604 ± 0.2798, the fractal dimension of a square mask is *D*_2_ = 1.596 ± 0.0460, and the lacunarity is *Λ*_2_ = 0.0402 [32]. The fractal dimension is much smaller, but the lacunarity is greater because the xerogels used in the current/implicated article have more voids/interstices, such that they are able to fix mercury in larger quantities. The material has a high porosity, but the pores are occupied with fixed Hg. In the current article, the fractal dimension of an SEM figure for a rectangular mask is *D*_3_ =1.668 ± 0.3127 and for a square mask is *D*_4_ = 1.615 ± 0.1445, and the lacunarity is *Λ*_3_ = 0.0526. The free, unoccupied pores increase the lacunarity value of the SEM figures of the tested materials.

## 3. Conclusions

This paper depicts some xerogel solid materials, equipped with a multitude of pores, that are statistically represented in their compositional structure, most frequently between the values of zero and 25 microns.

To correctly investigate the porous xerogel dynamic comportment, in vitro kinetic information was considered to be respected by an acoustic equation of propagation. Thus, in a fractal porous media, this fractional differential equation was successfully verified and solved with the help of Bessel functions.

Moreover, the fractal character was demonstrated by utilizing fractal analysis and it has been proven on the evaluated SEM pictures of porous xerogel compounds. The fractal parameters, namely the fractal dimension, the lacunarity, and the Hurst index, were calculated with considerable precision. 

The results of the numerical evaluation on the selected L SEM picture, effectuated with the aid of the fractal characterization software developed by the authors, demonstrated that the values of the fractal dimensions and the Hurst exponents were *FD*_1_ = 1.668 ± 0.312 and *H*_1_ = 0.332, calculated with a square mask. In addition, *FD*_2_ = 1.615 ± 0.1445 and *H*_2_ = 0.242, calculated with a rectangular mask. The lacunarity value of *Λ* = 0.0526 was the last fractal parameter computed.

## 4. Materials and Methods

### 4.1. The Xerogels Materials and Synthesis

The most important materials utilized for obtaining xerogels were the chitosan with an attenuated molecular mass, tri-ethylene glycol monomethyl ether in a 97% concentration, sodium hydride in a 95% concentration, phenothiazine in a 98% concentration, phosphorus V-oxychloride in a 99% concentration, and magnesium sulfate in a 99.5% concentration. All of these were acquired through the SA Company (Sigma-Aldrich Company, St. Louis, Missouri, United States), and the fractal dimension was calculated for the type of rectangular mask *SD*_2_ [33]. TEGylated phenothiazine means that the phenothiazine heterocycle is replaced by TEG (tri-ethylene glycol); more precisely, the phenothiazine kernel is equipped with a TEG link that strengthens it. Ultimately, we obtained viscous chitosan, and the results were verified with the help of a calibrated Ubbelohde viscometer [34,35]. A suite of three chitosan-founded hydrogels was obtained by the imination chemical reaction with a formyl derivative of tri-ethylene glycol–phenothiazine. Thus, the chitosan hydrogelation in the company of tri-ethylene glycol–phenothiazine aldehyde may occur due to the imine constituent parts along with the self-assemblage in cluster formations from reticulated nodes. 

Chitosan is composed of a long biopolymer chain of N-acetylglucosamine. Nanoscale chitosan has several advantages such as high antibacterial activity, broad spectrum of activity, as well as low toxicity to mammalian cells. Thus, chitosan was chosen for various reasons, many of them unrelated to the scope of its research in the present article. However, upon trying to manufacture it for other purposes, we realized its advantages in the currently investigated field, namely, the fact that it is an unsuspected resource in terms of it having a wide range of porosities.

Aerogel conditioning of the chitosan makes it possible to prepare porous solids of a significant specific surface. The increase in the chitosan concentration or the degree of acetylation decreases the specific surface of the synthesized chitosan gel. 

The fractal nature of the porous xerogel compounds and the structural attributes of these materials have been demonstrated by particularly highlighting their porous nature. These attributes were presented through the known analyses performed, such as the SEM surface analysis (see Figure 5) and FTIR spectra of xerogel samples (see Figure 6a), revealing that it was the best candidate. It is also among the few safe materials whose behavior can be associated with an equation for advanced continuum-type mechanics of porous media. It has a generally anisotropic, mathematical product-like fractal geometry. In addition, it could be easily verified as the same material; chitosan xerogel provides an exemplary answer to the solution of the fractional propagation equation type, in fractal porous media. This fact thus becomes the biggest advantage of its use.

### 4.2. Xerogel Characterization

Low-density xerogels with functional amines and exhibiting very attractive textural properties can be obtained by one-step procedures using low-cost commercial ethyl silicate 40 (ES) instead of TEOS. These materials have notably larger pores, and a larger overall pore volume and specific surface area than the corresponding gels prepared using TEOS or TMOS alone [36,37]. The structural properties of xerogels depend on the type of amino functional silane additive. The use of a compound containing a more reactive methoxy group (ATM), acting as a nucleation agent, resulted in xerogels with larger mesopores and a more robust structure, similar to the results observed before.

In order to conduct fair comparisons and exemplary discussions in which usual xerogels are involved, some chitosan xerogel explanation samples were processed in the analogous conditions as the hydrogels mentioned in Section 4. All of these were denoted with the letter F and a certain number of indices. In other words, the experimentally realized SEM images of the samples are now available, and represent images of several different xerogels, each having diverse degrees of porosity. In the entire discussion held in this article, we presented only the picture that proved to be the most representative, as shown in Figure 5 [38]. 

### 4.3. Morphology

The xerogels discussed have a sponge-like morphology and are a declared an absorbent substance, with few isolated pores but rather interconnected pores. Statistically speaking, they seem to have a heterogeneous distribution, but with massive tendencies to approach the limit of a uniform distribution, with the pores formed having a diameter from 2 to 35 μm (Figure 5). Whilst the data in the literature describes the pore diameter increase as a decrease in the crosslinking degree, the same tendency was not observed in the case of our specimens [39]. Certainly, the imination rank in the hydrogel stage was not sufficiently large to manage the final morphology; consequently, the water freezing before the lyophilization enacts the crucial role. Thereby, the displacement of the imination balance (stability) to the resulting item in the lyophilization period totally consolidated the morphology type modeled in the freezing phase. However, the morphology was affected by the different sublimation cost/fee of water/acetone crystals from gelid hydrogels. The reduced density and acetone freezing point caused its rapid sublimation in contrast to pure water, resulting in a hydrophobic phenothiazine component congestion and fashioning a visible thin film at the superficial level in the xerogel [40,41].

### 4.4. The Morphology Characterization Techniques of a Porous Xerogel

The morphology characterization techniques of the porous xerogel structure were carried out via surface analysis by SEM, spectral analysis by Fourier transform infrared spectroscopy (FTIR) and the energy-dispersive X-ray (EDX) analyzer. The EDX has been utilized for the identification of the individual chemical elements in the examined composition [42]. Later, the SEM images will be evaluated by fractal parameter calculation, including the fractal dimension, the Hurst index, and lacunarity. The high accuracy with which these fractal indicators were calculated is owing to the methods and the advanced computational software used. Thus, the box-counting method was used to calculate the fractal dimension (see Figure 10), with different algorithms for a square mask and rectangular mask. As a measure of the particular accuracy obtained, the standard deviation *SD*_1_ = ±0.3127, calculated with a square mask. Moreover, the standard deviation of the fractal dimension calculated for the type of rectangular mask was *SD*_2_ = ±0.1445. 

To calculate the lacunarity, the modified SEM figures were used, with the binary version of the picture and the mask with well-defined pore contours (see Figure 8a,b) [43]. In addition, Figure 9 presents the assessed voxels of the SEM picture, with a 3D eloquent voxels portrait of the gray-level amount on the Oz-axis, conforming with the position and number of pixels, labeled on the last two plane axes: the Ox-axis and Oy-axis, respectively. In fact, this represents the 3D vision of the lacunarity due to the clear distinction of the pores in a processed SEM image.

We can carry out the SEM analysis of the xerogel-type materials and if we apply the algorithm for calculating the fractal dimension, this will tell us if we have a uniform material without gaps or a lacunar material with pores of various sizes. This technique not only predicts but precisely tells us the surface morphology of the evaluated xerogel. In our opinion, this supposed generalization presented in this paper is the only one capable of carrying out appropriate comparisons and discussions on the evaluated materials. In conclusion, we can apply the fractal dimension analysis to any type of xerogel system. In the assumption of the correct use of the proposed methodology, by maintaining the proper measurement conditions in the laboratory, the experimental results can be easily reproducible and therefore generalized.

## Figures and Tables

**Table 1:** Fractal parameters values of L SEM image.

Index	*FD*_1_	*SD*_1_	*H*_1_	*FD*_2_	*SD*_2_	*H*_2_	Lacunarity
L	1.668	±0.3127	0.332	1.615	±0.1445	0.242	0.0526

## Data Availability

The data used to support the findings of this study cannot be accessed due to commercial confidentiality.
